# Grand-maternal smoking in pregnancy and grandchild’s autistic traits and diagnosed autism

**DOI:** 10.1038/srep46179

**Published:** 2017-04-27

**Authors:** Jean Golding, Genette Ellis, Steven Gregory, Karen Birmingham, Yasmin Iles-Caven, Dheeraj Rai, Marcus Pembrey

**Affiliations:** 1 Centre for Child and Adolescent Health, School of Social and Community Medicine, University of Bristol, Bristol, UK; 2 Centre for Academic Mental Health, School of Social and Community Medicine, University of Bristol, Bristol, UK

## Abstract

Although there is considerable research into the genetic background of autism spectrum disorders, environmental factors are likely to contribute to the variation in prevalence over time. Rodent experiments indicate that environmental exposures can have effects on subsequent generations, and human studies indicate that parental prenatal exposures may play a part in developmental variation. Here we use the Avon Longitudinal Study of Parents and Children (ALSPAC) to test the hypothesis that if the mother or father (F1) had been exposed to their own mother’s (F0) smoking during pregnancy, the offspring (F2) would be at increased risk of autism. We find an association between maternal grandmother smoking in pregnancy and grand daughters having adverse scores in Social Communication and Repetitive Behaviour measures that are independently predictive of diagnosed autism. In line with this, we show an association with actual diagnosis of autism in her grandchildren. Paternal grandmothers smoking in pregnancy showed no associations.

Autism and Autism Spectrum Disorder are terms given to children less able to interact with the world in the way that other children do. Autism spectrum disorders (ASD) are characterised by social-interaction difficulties, communication challenges and a tendency to engage in repetitive behaviours. Diagnosis is not simple and can depend on the experience and sensitivity of the examiner to the normal range of child behaviours, and the insistence of the parents for a diagnosis for their child. In consequence there has been increasing interest in the aetiology of the traits that contribute to the diagnosis^1^, on the assumption that genetic and/or environmental factors may influence the severity of one or more particular traits and thus the likelihood of a diagnosis. In support of this view is evidence that autistic traits are heritable and continuously distributed in the general population^2^ and that ASD and autistic traits are etiologically linked^3^.

Dramatically increasing reported prevalence of ASD^4^ may partly be a result of changing definitions and increasing awareness, but there is evidence that some of the increase is probably real^5^. This perceived increase has focussed attention on possible environmental exposures that may have increased over time. Although maternal smoking in pregnancy has been assessed many times in relation to ASD risk, with conflicting results^6^, there is a possibility that it may not be so much exposure during the pregnancy resulting in the affected child, but rather that of a preceding generation. There are many animal experiments that indicate that prenatal environmental exposures have effects on subsequent generations which are often sex-specific and via germline transmission, in line with human multigenerational observations^7^.

Over the last decade an increasing number of genetic variants and mutations, in both nuclear and mitochondrial DNA, have been associated with ASD risk^8^,^9^. These range from common to rare DNA sequence changes which may be inherited from a parent or arise *de novo* in gametes or in the early embryo. *De novo* point mutations in the nuclear genome usually arise in sperm and these increase with a man’s age leading to an interest in paternal age effects on ASD risk. Indeed, offspring of older men do have an increased risk of ASD and schizophrenia, but it seems that *de novo* mutations only account for 10–20% of this paternal-age-related increase^10^. The latter paper uses empirical epidemiological data to model several scenarios including the possibility that (an inherited) elevated liability to psychiatric illness may delay fatherhood; the authors conclude that ‘genetic risk factors shared by older fathers and their offspring are a credible alternative explanation to *de novo* mutations for risk to children of older fathers’. However, an inherited liability need not necessarily be solely genetic. It is possible that intergenerational effects of early-life exposures may also contribute to this liability to ASD.

Among humans there are few longitudinal data sets which can address the question as to whether parental prenatal exposure via a pregnant grandmother (F0) might have a biological influence on the development of the grandchild (F2). Grandmaternal smoking in pregnancy has been studied in the Avon Longitudinal Study of Parents and Children (ALSPAC), where an investigation into growth had shown that prenatal exposure of a parent to the smoking of their mother has a sex-specific effect on their own offspring’s birthweight and childhood growth measurements. The results have shown that, provided the mother herself does not smoke in pregnancy, the maternal grandmother’s prenatal smoking is associated with increased fetal growth in her grandsons^11^, and subsequent increases in lean mass, especially during adolescence, with this being reflected in increased strength of these boys^12^. In contrast, if the paternal grandmother had smoked when expecting the father and the mother had smoked in pregnancy, her grandsons had a smaller mean head circumference reflected in a reduced mean IQ compared to when the mother but not the grandmother smoked in pregnancy^13^.

The ALSPAC study has followed 14000 children from birth, identifying a number of traits at different ages. A detailed analysis of the predictive value of these traits to identify children with autism showed four to be independently associated^14^. The autistic traits comprised a social communication score, a speech coherence score, a sociability temperament scale, and a repetitive behaviour score. These four traits form the basis of this study together with the ~170 children with diagnosed autism. We assess whether extreme levels on any of the four traits are associated with either of the grandmothers smoking in the pregnancy resulting in the birth of the study parent. There are two pathways of possible influence of parental prenatal exposure to cigarette smoke on the study child: (a) via the maternal grandmother (F0) to the mother (F1) while *in utero,* thence to her study fetus (F2), and (b) via the paternal grandmother (F0) to the study father (F1) while he was *in utero* and thence to the study conceptus (F2).

## Results

### Relationships with trait outcomes

#### The Social Communication trait

**Figure 1:**
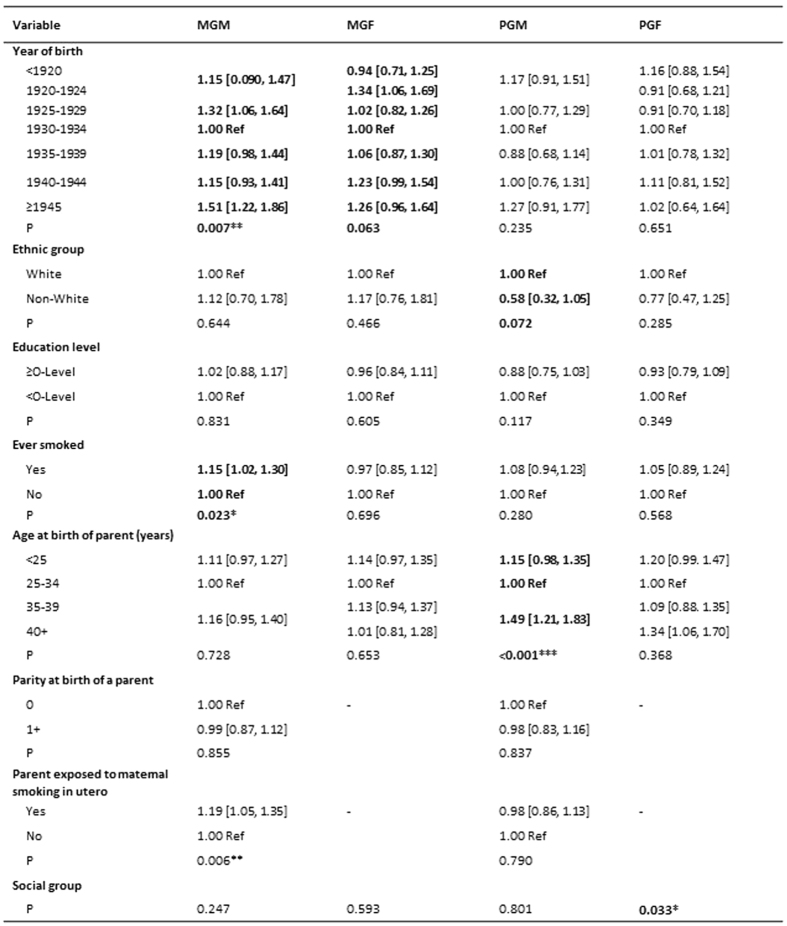
The Social Communication trait. The table shows the unadjusted odds [95% CI] of high scores on this trait at age 7 in relation to features of the grandparents.Variables with P < 0.10 are in bold. ^*^P < 0.05; ^**^P < 0.01; ^***^P < 0.001. MGM = maternal grandmother; MGF = maternal grandfather; PGM = paternal grandmother; PGF = paternal grandfather.

The relationships between characteristics of the grandparents of children with poor levels of social communication are shown in Fig. 1. The following factors were significant at the 0.10 level: maternal grandparents’ year of birth; maternal grandmother was a smoker; paternal grandmother was aged 35 or more at the birth of the study father, paternal grandmother’s ethnic group and paternal grandfather’s social group. In addition there was an increased odds for the maternal grandmother smoking during the pregnancy resulting in the mother [UOR 1.19; 95% CI 1.05, 1.35] (Fig. 1).

**Figure 2:**
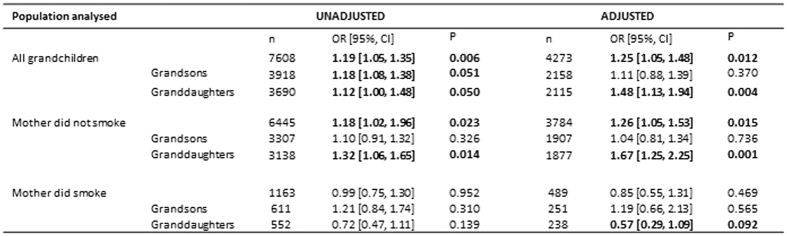
Unadjusted and adjusted odds ratios of maternal grandmother smoking prenatally in regard to high scores on the Social Communication trait. Variables with P < 0.10 are in bold.All adjusted variables take account of the year of birth of the maternal grandmother, the year of birth of the maternal grandfather, the age of the paternal grandmother when the study father was born, the social group of the paternal grandfather and the sex of the grandchild (where appropriate).

Based on the unadjusted relationships we recoded the years of birth variables as follows: maternal grandmother, before 1935, 1935–1944, after 1944; maternal grandfather, before 1940 and 1940 or later. These and all the other variables at P < 0.10 were taken into account when looking at the relationship between prenatal grandmaternal smoking and high scores on the Social Communication scale using logistic regression (Fig. 2). We did not include ethnic group as minority numbers were very small. The results of adjustment are shown in Fig. 2. Taking all children together the odds ratio for grandmaternal prenatal smoking increased from 1.19 [95% CI 1.05, 1.35] to 1.25 [1.05, 1.48] on adjustment. The unadjusted odds ratio was slightly higher for boys (1.18) than girls (1.12), and both were of borderline significance. However, on adjustment the association reduced for boys to 1.11 [0.88, 1.39] but increased for girls (1.48 [1.13, 1.94]; P = 0.004).

Further analysis separated out the risks to the children of mothers who had not themselves smoked in pregnancy. The unadjusted and adjusted odds were very similar to those for the whole population. However the split by sex of the grandchild demonstrates that the effects were more marked – unadjusted data showed ORs of 1.10 and 1.32 respectively, and these decreased and increased respectively such that the odds for boys was 1.04 [0.81, 1.34] and for girls 1.67 [1.25, 2.25] (P = 0.001). There was a significant interaction between the boys and girls.

Numbers of mothers smoking during pregnancy was relatively low, especially on adjustment. There was no suggestion of a grandmaternal prenatal smoking effect in this group (odds before and after adjustment were 0.99 and 0.85 respectively). However there were differing patterns with the sex of the child – for boys the odds changed from 1.21 to 1.19, whereas for girls the changes were from 0.72 to 0.57. The adjusted ORs by sex showed a statistically significant interaction. There was also a significant interaction among the girls between the children of mothers who smoked and those who did not.

#### The Speech Coherence Trait

**Figure 3:**
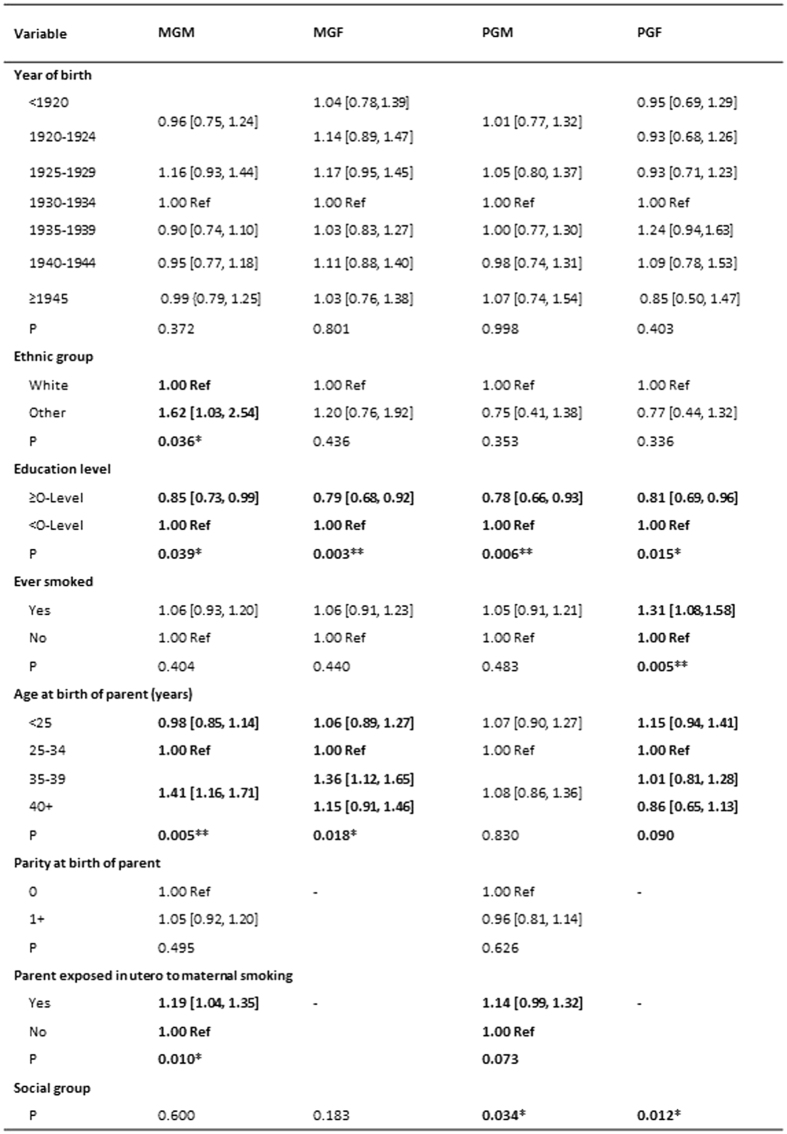
The Speech Coherence Trait. The table shows the unadjusted odds [95% CI] of poor coherence at age 9 in relation to features of the grandparents. Variables with P < 0.10 are in bold. ^*^P < 0.05; **P < 0.01; ***P < 0.0001.MGM = maternal grandmother; MGF = maternal grandfather; PGM = paternal grandmother; PGF = paternal grandfather.

The unadjusted odds ratios indicating the ways in which the prevalence of poor scores on the Coherence trait compare with those with higher scores is shown for the features of the children’s grandparents in Fig. 3. The results vary between the different grandparents, with the maternal grandmothers having an excess of non-white ethnicity, poorer education levels, older ages at the birth of the mother and, although there was no evidence of this grandmother having been a smoker during her lifetime, she was reported to have been more likely to have smoked during the pregnancy that resulted in the mother. In comparison there were fewer significant associations with the background of the maternal grandfather apart from a poorer educational level and an older age at the time of her birth. Neither of the maternal grandparents were more likely to have had occupations that indicated that they were socially disadvantaged.

**Figure 4:**
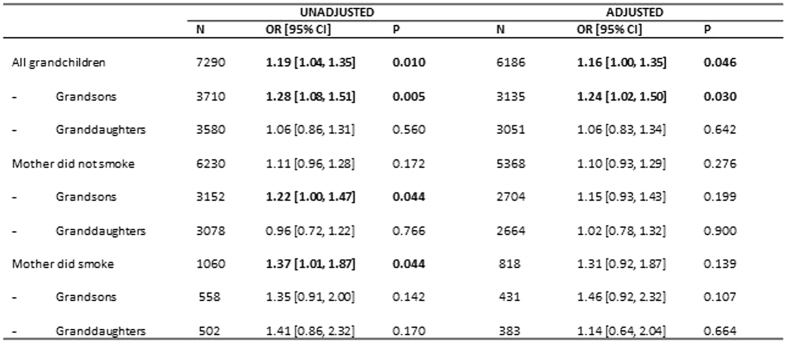
Unadjusted and adjusted odds ratios of maternal grandmother smoking prenatally in regard to poor scores on the Speech Coherence trait (all adjusted variables take account of the year of birth of the maternal grandmother, the year of birth of the maternal grandfather, the age of the paternal grandmother when the study father was born, the social group of the paternal grandfather and the sex of the grandchild (where appropriate). Variables with P < 0.10 are in bold.

In order to determine whether these significant features distinguishing the maternal grandparents explained the association with grandmaternal prenatal smoking, we categorised those maternal grandparent variables that had been statistically significant in the unadjusted analyses as follows: grandmother’s educational qualifications (high v low); grandmother’s age at the birth of the study mother (<35 v 35+), grandfather’s age at birth of study mother (<35 v 35+), grandmother’s ethnic status (white v rest) and put them into a logistic regression model; the grandmother smoking in pregnancy was then added. The results are shown in Fig. 4.

The odds of the grandchild having poor coherent speech reduced slightly on adjustment (from 1.19 [1.04, 1.35] to 1.16 [1.00, 1.35]) but retained statistical significance at P < 0.05. Subdivision by sex of the grandchild revealed a significant association for boys (AOR 1.24 [1.02, 1.50]) but not girls (AOR 1.06 [0.83, 1.34]). Subdivision by whether or not the mother herself smoked was not informative, although the adjusted risk to boys was always greater than the risk to girls. In contrast to the results for social communication, there were no significant interactions with sex.

Unadjusted associations of paternal grandparents whose grandchildren had poor speech coherence mirrored that of maternal grandparents in that they were less likely to have achieved the equivalent of O-level education, and the grandmother had been more likely to have smoked when pregnant with the study father. In comparison with the maternal grandparents, the paternal grandfather was more likely to have been a smoker, to have been relatively young when the study father was born, and both paternal grandparents were more likely to have come from poorer social circumstances as rated by their occupations (Fig. 3). The risk to the grandchild whose grandmother had smoked when carrying the father was of borderline significance (OR 1.14 [0.99, 1.32]), but was attenuated on adjustment for these variables (OR 1.09 [0.91, 1.30]).

#### The Repetitive Behaviour trait

**Figure 5:**
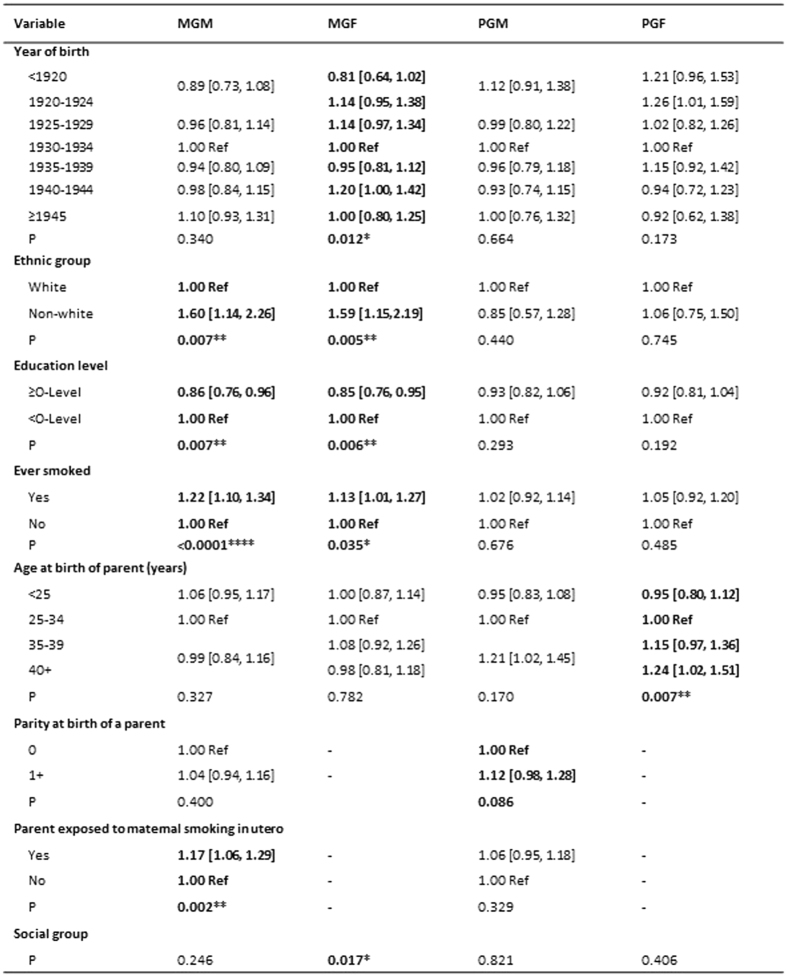
Repetitive Behaviours Trait. Variables with P < 0.10 are in bold. *P < 0.05; **P < 0.01; ***P < 0.0001; ***P < 0.001. MGM = maternal grandmother; MGF = maternal grandfather; PGM = paternal grandmother; PGF = paternal grandfather.The table shows the unadjusted odds [95% CI] of repetitive behaviour at age 5 in relation to features of the grandparents.

The way in which high scores on repetitive behaviour relate to features of the grandparents are demonstrated in Fig. 5. Most of the significant associations relate to the maternal rather than the paternal grandparents. These include the year of birth of the grandfather (with inconsistent variation), non-white ethnic origins of both, poorer education levels of both, and ever smoked for both. The unadjusted association between the maternal grandmother smoking in pregnancy and high score on repetitive behaviour was significant (P = 0.002). There was no indication that paternal grandmother smoking in pregnancy was associated with this trait in the grandchildren.

**Figure 6:**
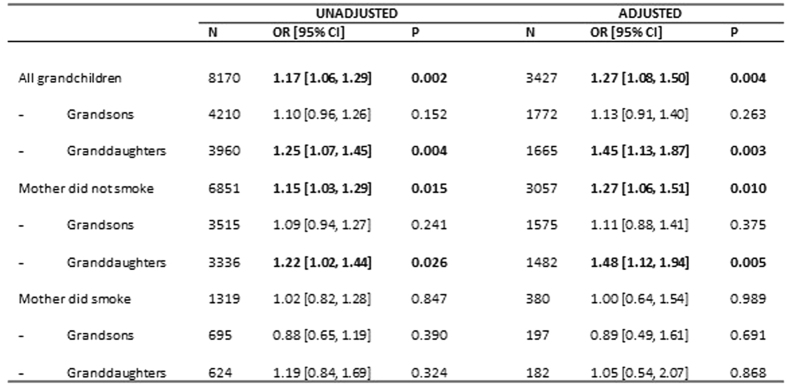
Unadjusted and adjusted odds ratios of maternal grandmother smoking prenatally in regard to high scores on the Repetitive Behaviours trait. All adjusted variables take account of the year of birth of the maternal grandmother, the year of birth of the maternal grandfather, the age of the paternal grandmother when the study father was born, the social group of the paternal grandfather and the sex of the grandchild (where appropriate).Variables with P < 0.10 are in bold.

Adjustment of grandmother smoking in pregnancy for grandmaternal ethnic group, educational level and social group of maternal grandfather, and ages of the paternal grandparents resulted in an increase in the odds for repetitive behaviour from 1.17 [1.06, 1.29] to 1.27 [1.08, 1.50], with strong relationships particularly for grand daughters (AOR 1.45 [1.13, 1.87] P = 0.003) compared with grandsons (AOR 1.13 [0.91, 1.40]). A similar pattern was shown for the risk to the grandchildren whose mothers had not smoked in pregnancy, but there was no discernible effect when the mother herself had smoked (Fig. 6). It should be noted that the goodness of fit for the adjusted model (using pseudo-R^2^) when the maternal grandmother smoking in pregnancy variable was added to the model increased by 3-fold, indicating that the grandmother’s smoking in pregnancy was a key grandparental influence on this trait.

#### The Sociability Trait

**Figure 7:**
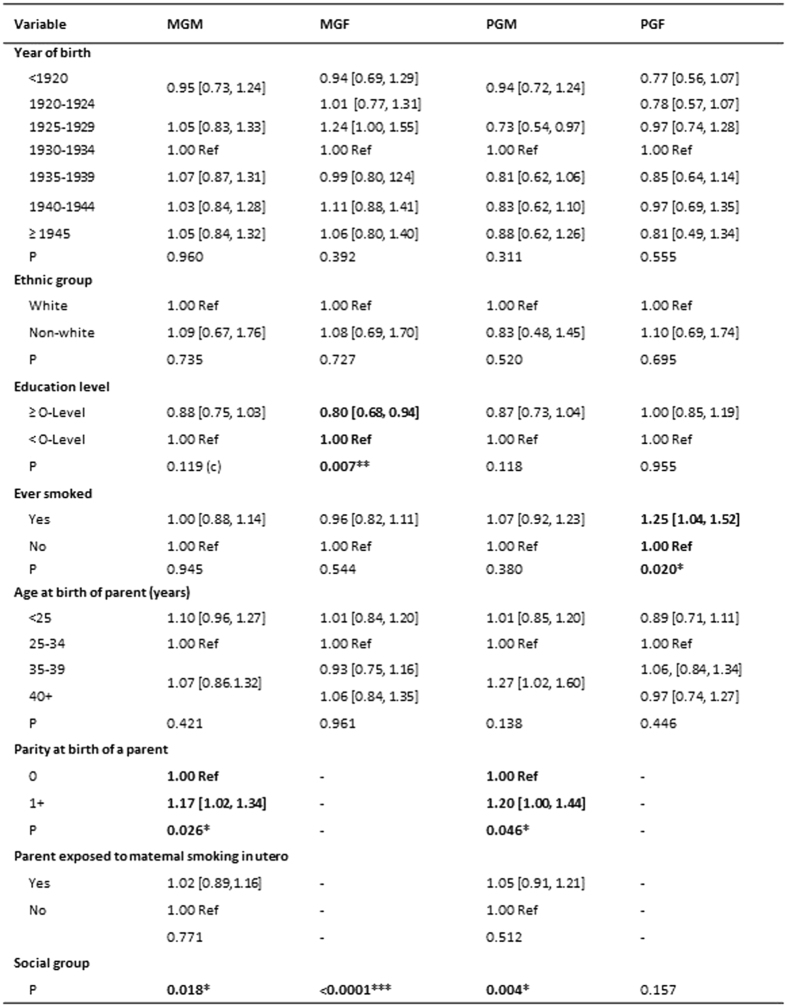
The Sociability Trait. Variables with P < 0.10 are in bold. ^*^P < 0.05; ^**^P < 0.01; ^***^P < 0.0001.MGM = maternal grandmother; MGF = maternal grandfather; PGM = paternal grandmother; PGF = paternal grandfather.The table shows the unadjusted odds [95% CI] of poor sociability scores on this trait at age 3 in relation to features of the grandparents.

The ways in which poor scores on the sociability trait vary with the characteristics of the grandparents is shown in Fig. 7. There were fewer significant features than found in the other traits – *viz* maternal grandfather’s education; paternal grandfather’s smoking habit, parity of the grandmothers when pregnant with the parents, and the social categorisations of both grandmothers and the maternal grandfather. There was no significant association with either the maternal or paternal grandmother smoking prenatally (OR 1.02 and 1.05 respectively). Consequently we did not analyse this trait further.

#### Relationships with diagnosed autism

**Figure 8:**
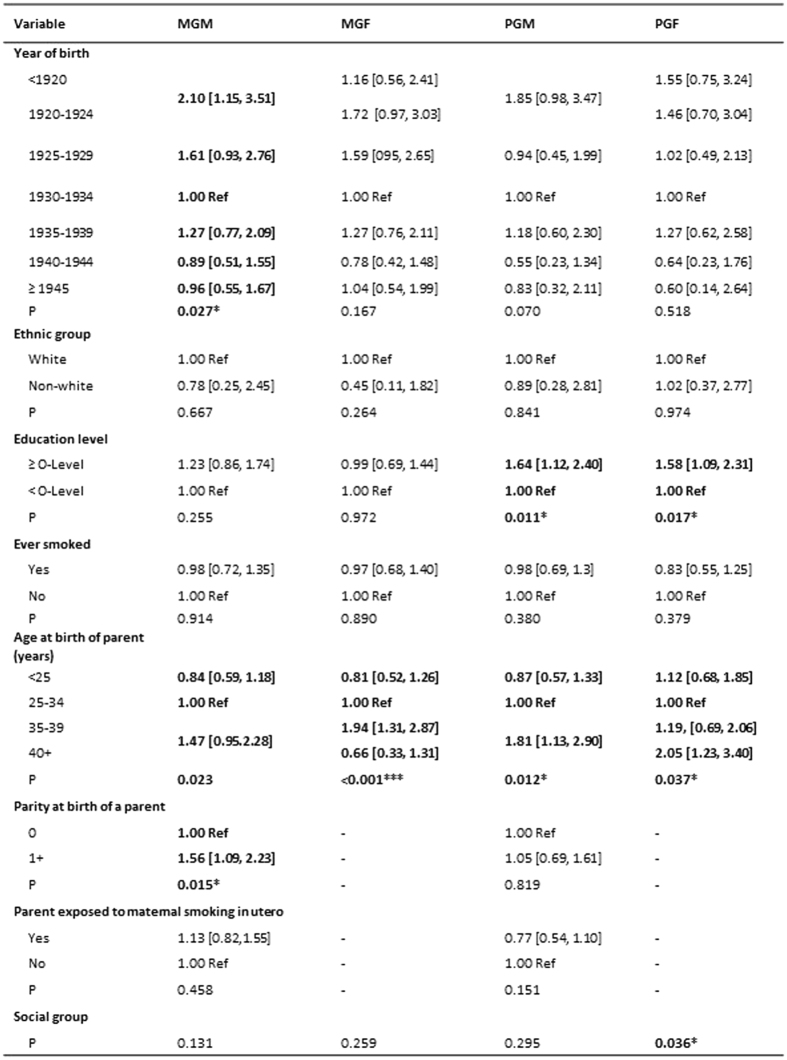
Diagnosed autism. The table shows the unadjusted odds [95% CI] of diagnosed autism in relation to features of the grandparents. Variables with P < 0.10 are in bold. ^*^P < 0.05; ^**^P < 0.01; ^***^P < 0.001.MGM = maternal grandmother; MGF = maternal grandfather; PGM = paternal grandmother; PGF = paternal grandfather.

Of the 212 offspring (F2) with possible or definite diagnosed autism, ~170 had information on at least one of the grandparents. Figure 8 demonstrates the variation between the unadjusted associations between the descriptions of the grandparents and the odds ratios for autism. The associations that were significant at P < 0.10 for the maternal grandparents comprised the year of birth of the grandmother, the ages of each grandparent at the birth of the mother, and the mother’s parity at the time of this birth. At this stage the association with maternal grandmother’s smoking in pregnancy was not significant at this level. However, knowing the biased ascertainment of autism in this population, we had decided prior to this finding that any analysis should take account of the social factors relating to the mother’s (F1) use of social housing, her education level and her locus of control as well as features relating to the maternal grandparents (F0).

**Figure 9:**
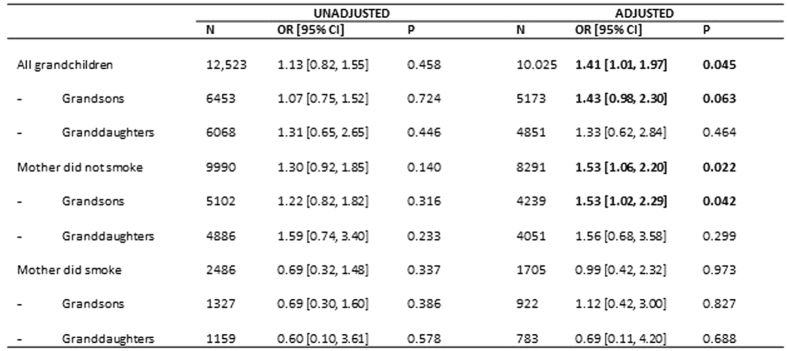
Unadjusted and adjusted odds ratios of maternal grandmother smoking prenatally in regard to diagnosed autism. All adjusted variables take account of the year of birth of the maternal grandmother, the ages of the grandparents when the study mother was born, the parity of the maternal grandmother, mother’s (F1) use of social housing, education and locus of control. [N.B. Parity was dropped from the subgroup analyses of mothers who smoked since there were no maternal grandmothers of parity 0 in this group]. Variables with P < 0.10 are in bold.

The results of these adjustments are shown in Fig. 9. The unadjusted association 1.13 [0.82, 1.55] increased on adjustment to 1.41 [1.01, 1.97]. Selection of the population where the mother herself (F1) did not smoke showed a greater adjusted OR (1.53 [1.06, 2.20]), with the associations for boys and girls being similar, although the association for grand daughters was not statistically significant due to the much smaller numbers involved (there were 4 times as many boys as girls with ASD).

For the populations for which the mother herself (F1) had smoked during pregnancy there was no indication of any association with maternal grandmother (F0) smoking in pregnancy. Nor was there any association with paternal grandmother smoking in pregnancy.

## Discussion

The aims of this study were to assess whether: (i) a history of either grandmother smoking in pregnancy was associated with any of the four behavioural-trait scores independently predictive of autism in the grandchild or with diagnosed autism in the grandchild; (ii) whether any such relationships varied with the sex of the study child, and (iii) whether the results depended on whether or not the study mother also smoked in pregnancy. We found that two of the four autistic traits in the grandchild (F2) were increased in prevalence if the maternal grandmother (F0) smoked in pregnancy especially if the mother herself (F1) did not herself smoke, but that diagnosed autism was also associated with the maternal grandmother smoking in pregnancy.

For two of the traits (Social Communication and Repetitive Behaviour) we have shown that grand-daughters were much more affected than grandsons, and that the associations were particularly apparent if the mother herself did not smoke in pregnancy. The associations tended to increase in effect size after adjustment for social and biological factors of the grandparents. This is not the first time that a sex-specific association between grandmother’s prenatal smoking and grandchild outcome has been shown. Previous studies from ALSPAC have used child growth as outcomes^11^,^12^. These demonstrated strong positive effects on fetal growth when the maternal grandmother smoked in pregnancy – the associations were found with grandsons but not grand daughters. During childhood these boys continued to put on weight, especially lean weight. When the paternal grandmother smoked in pregnancy, there was little effect on fetal growth of the grandchild, but the grandchildren grew taller and larger especially if they were girls. Clearly the relationships between grandmother’s prenatal smoking and grandchild’s growth is complex, especially as the effect sizes tended to increase as the grandchild went through adolescence. For this reason in this study, we extended the study to look at the social communication trait at age 16, but found no difference in effect size compared with the relationships at age 7 (data not shown).

These results are intriguing. There remains the question as to whether they are the result of confounding that has not been taken into account. This seems unlikely since on allowance for the demographic and biological factors, the effect sizes increased rather than decreased. If there was a systematic failure in the number of characteristics taken into account, one would expect similar results for paternal grandmother smoking as for maternal grandmother exposure; one would also expect the same results for boys as for girls. We have shown that, after adjustment, cases of diagnosed autism overall were similarly associated with maternal grandmother’s smoking in pregnancy, and similarly increased risk when the mother (F1) did not smoke, as found with two of four different traits predictive of ASD. Such findings make a causal connection more plausible, although the mechanism warrants further investigation. Unfortunately the numbers of children with diagnosed autism were not sufficient to assess whether there were sex-specific results as found with the two traits.

We only allow for features of the grandparents rather than the parents when considering the traits, since parental attributes may well be on the causal pathway. Indeed a further exercise for this study in the future is to determine whether there are features of the personality and attitudes of the mother that may explain the associations between her exposure in utero and her child’s autistic traits. For diagnosed autism, we are aware that although we used a variety of sources to identify the cases, there are consistent gaps – particularly where the mother had a low level of education, lived in public housing and/or had an external locus of control orientation. We considered it important to take these factors into account because they were likely to have resulted in the lack of a diagnosis even though the criteria were present. Interestingly, only when these factors were taken into account was the association with grandmother’s prenatal smoking revealed.

One statistical criticism may be that we did not allow for missing data. The decision not to do this concerned our doubts as to whether the data were missing at random. We strongly suspect not, and have therefore not employed techniques such as MICE. However it is important to note that the results shown were mostly apparent in both the unadjusted and adjusted results.

There are a number of strengths to this study. (a) The population was defined by expected date of delivery and geographical area of residence; it is therefore not biased by likelihood of having autistic traits. (b) A large number of scales were assessed to determine the likelihood of which were able to best predict ASD; this process involved all children for whom the data were available. (c) The data concerning features of the grandparents were collected during the pregnancy resulting in the grandchild, therefore prior to any suggestion that there was anything amiss with the behaviour or development of the child. (d) All parents were invited to complete all questions in the frequent questionnaires they received, not just those relevant to their child.

However there are a number of limitations: (1) We are relying on the accuracy of the reports by the study parents concerning their parents, and this would have been difficult for those who had difficult childhoods; they were encouraged to contact relatives wherever possible to fill in gaps in their own knowledge (an advantage of having the leisure to complete a questionnaire in their own home). (2) The results are mainly relevant to white grandparents living in Britain, the numbers were too small to subdivide the analysis into different minority ethnic backgrounds. (3) The study was not originally planned to look at autism as, at the time of planning (1988) the prevalence was thought to be so low as to suggest that no more than 10 cases might be included in the study. Consequently sets of trait questions were not designed as measures of autistic traits but rather to identify the child’s performance in regard to a large number of attributes at different ages; regression analyses had identified those related to social communication, coherent speech, repetitive behaviour and sociability as being independently predictive of ASD within the ALSPAC study^14^. (4) Similarly the questions on abnormal and repetitive behaviour were used post hoc to define an autistic trait, and could be criticised for this.

Some may consider the fact that we have not corrected for the number of tests carried out as a criticism, but it should be remembered that we were testing a specific hypothesis concerning the possibility of prenatal smoke exposure to the F1 generation having an association with autism and autistic traits in generation F2. We had stipulated sex differences and differences between the offspring of women who did and did not smoke. We used nine tests for each of the five outcomes, and demonstrated 15 adjusted associations at P < 0.05, compared with 2 expected if the results occurred by chance.

There are two plausible candidate mechanisms for the observed association of ASD risk with maternal prenatal tobacco exposure; transmission of damage to mitochondrial DNA (mtDNA) or epigenetic inheritance from one generation to the next. Maternal smoking in pregnancy causes mtDNA damage to the newborn^15^ and the transmission of mtDNA variation particularly heteroplasmic mutations (i.e. coexistence of normal mtDNA and mtDNA with pathogenic mutations in the same cell) is increased in ASD^9^. Mitochondrial transmission across the generations is exclusively via the mother, so is compatible with our observed associations between maternal prenatal tobacco exposure and adverse scores on Social Communication and Repetitive Behaviour measures in her granddaughters. Typically, a mother transmitting heteroplasmic mtDNA mutations is asymptomatic. A maternal grandmother smoking in the latter part of pregnancy could result in direct tobacco effects on both the developing oocytes of her female fetus, and the recombining chromosomes contained therein that are destined for any grandchildren. There is evidence that mitochondrial transmission acts as a sex-specific selective sieve with mutant mtDNA being better tolerated in females compared to males^16^. How this relates to the sex-specificity observed in this study is unclear.

Epigenetic regulation of gene expression through DNA methylation, histone modification and non-coding RNAs is recognised as part of an organism’s response to environmental exposures, but how elements of this epigenetic response may be transmitted to the next generation(s) is largely unresolved.

Epigenetic transmission in relation to ASD risk has been explored within high risk families using human sperm that are more accessible than oocytes^17^. The authors conclude that epigenetic (DNA methylation) differences in paternal sperm may contribute to autism risk in offspring based on the Autism Observational Scale for Infants at 12 months of age. To our knowledge, there are limited published data on human *multigenerational* studies of epigenetic adjustments in relation to maternal smoking, but there are large DNA methylation studies of children exposed to maternal smoking as a fetus. A recent meta-analysis^18^ revealed differential methylation at several genes previously associated with ASD. These include the DLGAP2 (SAPAP2) gene that encodes a protein involved in the molecular organization of synapses and in neuronal cell signalling and is known to be linked to ASD^19^,^20^; and also the neuropilin-2 (NRP2) gene, polymorphisms of which have been associated with autism^21^. The association of child DNA methylation with maternal smoking in pregnancy has been studied in ALSPAC with a focus on the persistence of the differential methylation from newborn to age 17 years^22^ One such persistent altered methylation pattern involved the CNTNAP2 gene that encodes a neuronal transmembrane protein member of the neurexin superfamily involved in neural-glia interactions. A common variant of CNTNAP2 is an established risk factor for autism^23^,^24^. Indeed CNTNAP2 variants are associated with some of the traits that predict ASD risk used in this study^14^, although another study did not find this^25^. Whilst the above associations lend support for the idea that smoking may induce (directly or indirectly) epigenetic changes in fetal genes relevant to ASD risk, they do little to clarify the nature of the positive association with ASD risk in the subsequent generation. The epigenetic response is likely to be complex. Smoking is associated with DNA damage^26^ and this in turn may induce the production of non-coding (micro) RNAs that can be transmitted to subsequent generations to target several genes relevant to ASD for silencing^27^.

We have used exposure of the parent to smoking in utero for our studies of intergenerational effects for two reasons. First this is a habit that is easily remembered; and second, cigarette smoking in pregnancy is known to have an adverse effect on child development, and on DNA methylation – and an effect on the next generation is theoretically possible. The analyses were undertaken to ascertain whether there might be sex-specific associations between autistic traits and parental exposure to smoking in utero, but without prior hypotheses as to which grandmother or child’s sex might be involved; consequently it is particularly important that these associations be confirmed in other studies.

## Methods

### The ALSPAC study

The data used in these analyses were collected as part of the Avon Longitudinal Study of Parents and Children (ALSPAC), which was designed to assess the ways in which the environment interacts with the genotype to influence health and development^28^. Pregnant women resident in the study area in south-west England with an expected date of delivery between 1^st^ April 1991 and 31^st^ December 1992, were invited to take part. About 80% of the eligible population did so^29^^29^. The initial ALSPAC sample consisted of 14,541 pregnancies; of these, 14,472 had known birth outcomes: 14,062 were live births and 13,988 were alive at 1 year. Information was collected using a variety of methodologies including self-completion questionnaires sent to study mothers, fathers, teachers and the study child, direct examination under standardised conditions, and linkage to educational data from the school system.

### Data Availability

The study website contains details of all the data that are available through a fully searchable data dictionary:

[http://www.bris.ac.uk/alspac/researchers/data-access/data-dictionary/].

Data can be obtained by bona fide researchers after application to the ALSPAC Executive Committee (http://www.bristol.ac.uk/alspac/researchers/access/).

### The Exposures

The pregnant study mothers and their partners (F1) were sent a number of questionnaires during pregnancy. These elicited information on their smoking histories and those of their parents (i.e. the study grandparents (F0)). If they reported that their mothers (F0) had smoked, they were each asked whether their mothers had smoked when expecting them – and, if so, were given the responses yes/no/don’t know from which to select. Thus the parents who replied ‘don’t know’, had a mother who smoked but the parent was unsure whether she had smoked during her pregnancy. We have analysed these data assuming that these women did smoke during pregnancy. This assumption has been validated by demonstrating that the mean birthweights of this group of study mothers were reduced when compared with those who reported that their mother had definitely not smoked in pregnancy^11^.

### Possible confounders

Other information collected on the four study grandparents included the years in which they had been born, their ages when the study parent was born, their social group (based on their occupations), their educational qualifications (grouped into low level of qualifications (or none), and the equivalent of O-level or higher), ethnic group (grouped as white and all other), and (for grandmothers only) parity – i.e. whether the study parent was the first or later birth to that grandmother. All of these variables were considered as possible confounders.

### The autistic spectrum traits

We have used the four independent trait predictors of ASD identified by Steer and colleagues^14^ as being the best of 93 trait measures to be independent predictors of autism diagnosed using ICD-10 criteria. These are described below. All have a continuous distribution, but only the more abnormal scores were shown to be associated with diagnosed autism. We have therefore analysed the traits as binary variables using a cut of approximately 15% to identify the high risk group.

### The Social Communication Trait

We used the 12-item Social and Communication Disorders Checklist (SCDC), which was developed by Skuse and colleagues^30^ specifically as a screening tool to identify children at high risk of autism. They showed that the internal consistency was excellent (0.93) and the test-retest reliability was high (0.81). The method was developed on clinical samples, and when later tested on the ALSPAC population at age 7.7 years the high end of the scale was shown to predict a variety of adverse outcomes, but was most specific for ASD^31^. Further research showed that the measure was fairly stable over time^32^.

For the present analysis we have used the prorated score, which was calculated when any items were missing a response by using the average of the items that had been answered by the individual (2.7% of the population, almost all of whom had just one item missing). If all items were missing, the score was put to missing. The measure ranged from 0 to 24, the higher the score the more impaired was the child’s social cognition. The distribution was skewed with a long upper tail; 16.8% had a score of over five and comprise the abnormal group for the present analyses.

### The Speech Coherence Trait

At age 9 the study mother completed a questionnaire on the speech of her child (F2) which included 7 of the 9 scales of the first version of the Children’s Communication Checklist (CCC)^33^. This checklist was designed to assess aspects of communication that are not readily assessed by conventional standardised tests including aspects of speech and syntax, as well as pragmatic aspects such as over-literal interpretation of stereotyped language. Although the CCC was initially designed to identify pragmatic difficulties, it has been shown to be good at discriminating a wide range of language and communication problems from typical development^34^. Analyses of traits predictive of autism in ALSPAC showed that the Coherence scale performed better than the other CCC scales^14^, and consequently that is used here. The scale comprised 8 items (e.g. ‘It is sometimes hard to make sense of what she is saying because it seems illogical or disconnected’ or ‘She has difficulty in telling a story, or describing what she has done in a sequence of events’). The score ranged from 20 to 36, with higher scores indicating more typical behaviour. The score had a skewed distribution. The lower tail used in this analysis comprised those children scoring <34 points, which identified 15.6% of the population having poor speech coherence.

### The Repetitive Behaviour Trait

This scale was developed from the answer to four questions in the questionnaire sent to the mother at 69 months; these were as follows: ‘How often does he/she (a) repeatedly rock his head or body for no reason; (b) have a tic or twitch; (c) have other unusual behaviour’; and (d) ‘Does he/she stumble or get stuck on words, or repeat them many times? (e.g. I I I I want a sweet)’? The responses to each question were coded as: ‘often/always = 3; sometimes = 2; never = 1′ and summed. The resultant scale had a range from 4 to 12, with 22% scoring 5 and only 6% scoring more than 5. Thus it was impossible to approximate to a 15% cut-off; we therefore used >4 as our abnormal group.

### The Sociability Temperament Trait

The questionnaire concerning the child (F2) sent to the study mothers when the child was 38 months of age included the 20 questions of the EAS Temperament scale^35^, and measured four traits – emotionality, activity, shyness and sociability – each based on the answers to 5 questions. The range of the Sociability sub-score was from 5 to 25 and the frequency distribution was approximately normal, a high score indicating a high level of sociability. The prorated scale was calculated for missing values as in the scales mentioned above. We then selected the lowest 11.4% of the children for our analyses (score <15) as being the nearest to 15%.

### Identification of diagnosed autism

In order to identify the children (F2) with diagnosed autism we used the following sources: (a) a review of all children given a statement for special educational provision in the Avon area to identify children diagnosed as on the autism spectrum using the ICD-10 criteria^36^; (b) the mother’s answer to the question ‘Have you ever been told that your child has autism, Asperger’s syndrome or autistic spectrum disorder’ at age 9; (c) classification by the educational system as requiring special educational needs because of an autism spectrum disorder by age 16; (d) text responses to any question on diagnoses given to the child in questionnaires from 6 months to 11 years; (e) ad hoc letters from parents to the Study Director. We considered that no one of these sources would be adequate, and so used all, and monitored the overlaps. This method identified 212 offspring with a reported (n-192) or possible (n = 20) diagnosis of autism (170 boys and 42 girls); of these 174 had information collected prospectively before birth, including details of the grandparents. These form the basis of the study of those with diagnosed autism.

In order to test the likelihood of including a high proportion of the true cases regardless of whether the parents had been given a diagnosis, we used the maternal responses to a set of questions from the DAWBA questionnaire answered at 91 months^37^ designed to identify an autism spectrum disorder. All were already identified among the 212 already identified. However this validation exercise could only be carried out among the participants who continued to complete questionnaires. We are aware that there will be a proportion who have not been diagnosed and that these are proportionately more likely to be among the socially deprived who were less likely to answer the questionnaires.

### Statistical Analyses

Coding and statistical methods were performed in accordance with the relevant guidelines and regulations. All methods involving human participants were performed in accordance with the relevant ethical guidelines. For each binary outcome we performed a separate set of logistic regression analyses – first looking at the unadjusted odds ratios (OR) and 95% confidence intervals (CIs) of each of the features of the grandparents as outlined in the potential confounders section above. We then selected all variables that were statistically significant at P < 0.10 to include in logistic regression analyses. The results were then investigated to determine whether there were differences in the results if the mother herself smoked prenatally, and whether there were differences between the results for boys and girls.

### Ethical approval

For the study was obtained from the ALSPAC Law and Ethics Committee and the Local Research Ethics Committees. Data were obtained from study parents voluntarily, and the relevant Committees deemed that completion of questionnaires and return by post comprised tacit agreement to take part in the study. Signed informed consent was obtained for all studies involving biological samples.

## Additional Information

**How to cite this article:** Golding, J. *et al*. Grandmaternal smoking in pregnancy and grandchild’s autistic traits and diagnosed autism. *Sci. Rep.* **7**, 46179; doi: 10.1038/srep46179 (2017).

**Publisher's note:** Springer Nature remains neutral with regard to jurisdictional claims in published maps and institutional affiliations.
